# Team Prenotification Reduces Procedure Times for Patients With Acute Ischemic Stroke Due to Large Vessel Occlusion Who Are Transferred for Endovascular Therapy

**DOI:** 10.3389/fneur.2021.787161

**Published:** 2022-01-03

**Authors:** Lars-Peder Pallesen, Simon Winzer, Christian Hartmann, Matthias Kuhn, Johannes C. Gerber, Hermann Theilen, Kevin Hädrich, Timo Siepmann, Kristian Barlinn, Jan Rahmig, Jennifer Linn, Jessica Barlinn, Volker Puetz

**Affiliations:** ^1^Department of Neurology, Dresden NeuroVascular Center, Carl Gustav Carus University Hospital, Technische Universität Dresden, Dresden, Germany; ^2^Carl Gustav Carus Faculty of Medicine, Institute for Medical Informatics and Biometry, Technische Universität Dresden, Dresden, Germany; ^3^Institute of Neuroradiology, Dresden Neurovascular Center, Carl Gustav Carus University Hospital, Technische Universität Dresden, Dresden, Germany; ^4^Department of Anesthesiology, Carl Gustav Carus University Hospital, Technische Universität Dresden, Dresden, Germany

**Keywords:** stroke, thrombectomy, work-flow, large-vessel occlusion, telemedicine

## Abstract

**Background:** The clinical benefit from endovascular therapy (EVT) for patients with acute ischemic stroke is time-dependent. We tested the hypothesis that team prenotification results in faster procedure times prior to initiation of EVT.

**Methods:** We analyzed data from our prospective database (01/2016–02/2018) including all patients with acute ischemic stroke who were evaluated for EVT at our comprehensive stroke center. We established a standardized algorithm (EVT-Call) in 06/2017 to prenotify team members (interventional neuroradiologist, neurologist, anesthesiologist, CT and angiography technicians) about patient transfer from remote hospitals for evaluation of EVT, and team members were present in the emergency department at the expected patient arrival time. We calculated door-to-image, image-to-groin and door-to-groin times for patients who were transferred to our center for evaluation of EVT, and analyzed changes before (–EVT-Call) and after (+EVT-Call) implementation of the EVT-Call.

**Results:** Among 494 patients in our database, 328 patients were transferred from remote hospitals for evaluation of EVT (208 -EVT-Call and 120 +EVT-Call, median [IQR] age 75 years [65–81], NIHSS score 17 [12–22], 49.1% female). Of these, 177 patients (54%) underwent EVT after repeated imaging at our center (111/208 [53%) -EVT-Call, 66/120 [55%] +EVT-Call). Median (IQR) door-to-image time (18 min [14–22] vs. 10 min [7–13]; *p* < 0.001), image-to-groin time (54 min [43.5–69.25] vs. 47 min [38.3–58.75]; *p* = 0.042) and door-to-groin time (74 min [58–86.5] vs. 60 min [49.3–71]; *p* < 0.001) were reduced after implementation of the EVT-Call.

**Conclusions:** Team prenotification results in faster patient assessment and initiation of EVT in patients with acute ischemic stroke. Its impact on functional outcome needs to be determined.

## Introduction

The benefit from endovascular therapy (EVT) for treatment of stroke due to intracranial large vessel occlusion is time dependent (1, 2). In patients who qualify for EVT in the neuro-interventional hospital, each 30 min increase of CT-to-reperfusion time is associated with a nearly 10% decreased chance to achieve a favorable outcome (3). Moreover, with each second of delay between arrival to groin puncture, the patient loses 2.2 h of healthy life-time (4).

Therefore, an efficient workflow should be paramount to reduce in-hospital treatment times (3, 5). The European recommendations on organization of interventional care in acute stroke (EROICAS) advise that imaging to treatment time (image-to-groin) should be <30 min and always <90 min (6). The Guidelines of the American Heart Association (AHA) recommend achieving a door-to-imaging time of <20 min but do not specify time goals in EVT patients (7). A multi-society (including the European Stroke Organization and World Stroke Organization) consensus statement proposed that 75% of patients being evaluated for EVT should have a door-to-imaging time of 30 min or less with high volume centers expected to achieve this in 12 min (8).

Telestroke networks have been shown to facilitate access of ischemic stroke patients to EVT by means of a drip-and-ship model with comparable results regarding safety and outcome (7, 9, 10). Furthermore, the transfer in the network itself is safe with medical interventions necessary in only a minority of patients (11). In contrast to patients who are directly admitted via ambulance, patients who are transferred from remote hospitals can be announced prior to arrival at the angiography site, leaving the staff with considerable time for preparation. However, due to potential long distances of travel between the community hospital and the stroke center and the possibility of transfer delays, patient admission to the angiography site might vary considerably (11, 12).

The objective of our study was to test whether the implementation of a semi-automated pre-notification procedure can lead to faster in-hospital treatment times of stroke patients who are transferred for EVT in a telestroke network.

## Materials and Methods

### Patients and Study Protocol

We performed a retrospective analysis of prospectively collected data on adult patients who were screened for EVT at our center. This EVT registry was established in January 2016 and contains detailed information on patients' demographics, vascular risk factors, neuroradiological imaging and stroke scores including National Institute of Health Stroke Scale (NIHSS) score and modified Rankin Scale (mRS) score. Admission non-contrast CT (NCCT) ASPECTS score, baseline status of the cerebral and pre-cerebral vasculature and reperfusion status after EVT (classified by modified Treatment in Cerebral Infarction [mTICI] score) were prospectively assessed by neuroradiologists. We defined mTICI scores of 2b or 3 as successful reperfusion (13). NCCT or magnetic resonance imaging (MRI) was routinely performed in all patients 24 h after EVT and additionally in case of clinically relevant neurological worsening. Furthermore, comprehensive data regarding treatment times including door-to-image, image-to-groin puncture, door-to-groin puncture, onset-to-groin puncture and groin-puncture-to-reperfusion times were collected. Treatment times before establishment of the prospective EVT registry were derived retrospectively based on electronic chart review.

During the study period, all transferred patients received repeated imaging with NCCT and CT angiography (CTA) after arrival at our center to assess the extent of early ischemic changes with ASPECTS and recanalization status. We also performed perfusion CT (CTP) in patients with late time window or in unclear cases. The decision whether to perform EVT was based on current guidelines and institutional protocols. Depending on clinical aspects we performed EVT under conscious sedation or general anesthesia with routine perioperative management by an anesthetist (14). General anesthesia was preferentially applied if patients had reduced level of consciousness, were non-cooperative or agitated or suffered from cardio-respiratory instability. After EVT, patients were immediately transferred to our intensive care unit or stroke unit for further treatment.

We assessed functional outcomes at 3 months by standardized telephone interview to determine the modified Rankin Scale (mRS) score (15). We defined favorable functional outcome at 3 months as modified Rankin Scale (mRS) scores of 0–2.

### Telestroke Network and Selection Criteria

**Figure 1:**
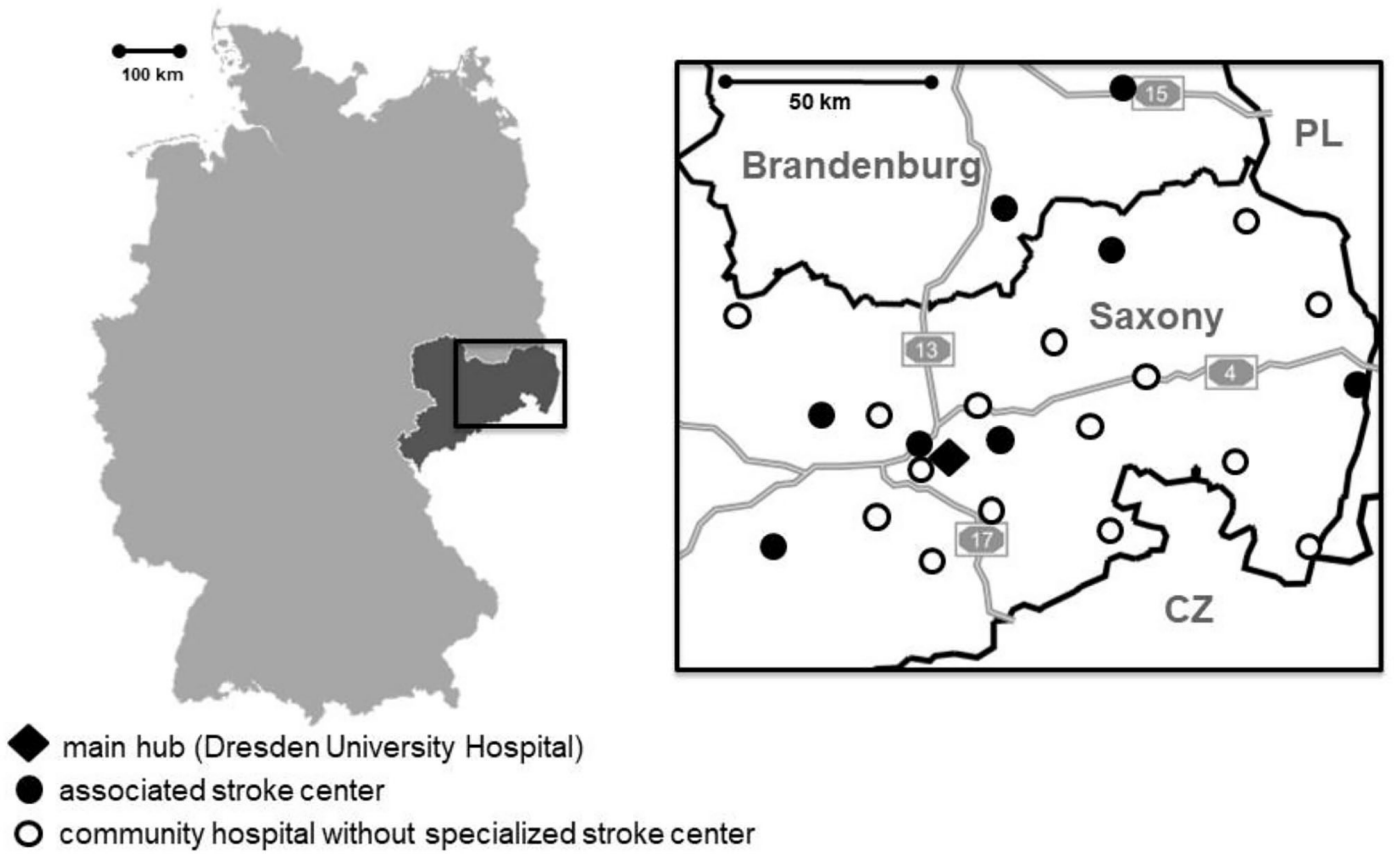
Left: map of the Federal Republic of Germany; the state of Saxony is highlighted in dark gray. Right: Map of our telestroke network in Eastern Saxony and Southern Brandenburg; dark lines indicate state and country borders; light gray lines with numbers indicate federal highways (*Autobahn*); CZ indicates Czech Republic; PL, Poland.

Our stroke network provides stroke expertise for the eastern part of the German state of Saxony and the southern part of the German State of Brandenburg (Figure 1). Approximately 2.4 million people reside in this area. During the study period, 15 community hospitals without a neurology department were part of the telestroke network. Further eight hospitals with dedicated neurology departments and certified stroke units but without or with limited EVT facilities were also part of this network. At the Dresden Neurovascular Center, an experienced stroke fellow is available 24/7 for teleconsultations and for organization of patient transfer and EVT from affiliated neurology departments.

To analyze the effect of the EVT-call on treatment times, we included all patients who were treated with EVT from January 2016 to February 2018 after transfer from remote hospitals without EVT capability in our stroke network. We excluded patients who were directly admitted to our emergency department.

### EVT-Call

We established an EVT-call as a phone-based semi-automated pre-notification procedure in July 2017 in order to shorten door-to-groin puncture times in our hospital:

EVT-call 1: After teleconsultation of the stroke fellow with the colleague at the remote hospital and decision to transfer the patient for EVT, the stroke fellow informs a central dispatcher, who is a member of the 24/7 staffed central communications office of our hospital, that a patient is to be expected. The dispatcher informs the interventional neuroradiologist, stroke neurologist, anesthetist, registered nurse in the emergency department, CT technician and angiography technician via a pre-recorded message on Digital Enhanced Cordless Telecommunications (DECT) telephones. The EVT-call 1 contains information on patients' sex and age as well as the name of the remote hospital.

EVT-call 2: After the patient transfer has started (i.e., time-point of departure of helicopter or ambulance), the physician at the remote hospital informs the stroke fellow that the patient has departed. The stroke fellow then sets up the EVT-call 2 to announce the transfer mode (helicopter or ambulance), the expected time of arrival and if the patient has been intubated prior to transfer. The expected time of arrival is determined according to distance and means of transportation based on prior transfer times. By communicating with the emergency service control center, the stroke fellow is able to ensure that the expected time of arrival is upheld, if necessary.

Before implementation of the EVT-call, the stroke neurologists telephonically informed the interventionalist to discuss eligibility for EVT on an individual basis. During on-call periods, the interventional neuroradiologist routinely assessed images from home and came into hospital if a patient was EVT eligible. With the implementation of the EVT-call, all team members were present in the emergency department at the expected time of arrival to streamline patient assessment and treatment decision making whether to perform EVT. If the patient was intubated prior to transfer a team consisting of one anaesthesiologist and one specialized nurse was present on patient arrival. In non-intubated patients, the anesthetist remained on stand-by and was called immediately after the decision for EVT was made. In general, we tried to avoid intubation for patient transfer in our stroke-network (11).

### Statistical Analysis

We applied non-parametric statistics because time-to-event data is often right-skewed. Continuous and non-continuous variables are presented as median [interquartile range, IQR] and percentage. Statistical comparisons were performed using *T*-Test, Fisher's exact test (two-sided), Mann-Whitney-*U*-test and non-parametric analysis of variance ANOVA, where appropriate (16). We considered a *p*-value < 0.05 as statistically significant for all analyses. Statistical analyses were performed using the software packages SPSS 23.0 and R 4.0.5 (IBM Corporation, USA and R Foundation [http://www.r-project.org], respectively).

## Results

### Patient Population

**Figure 2:**
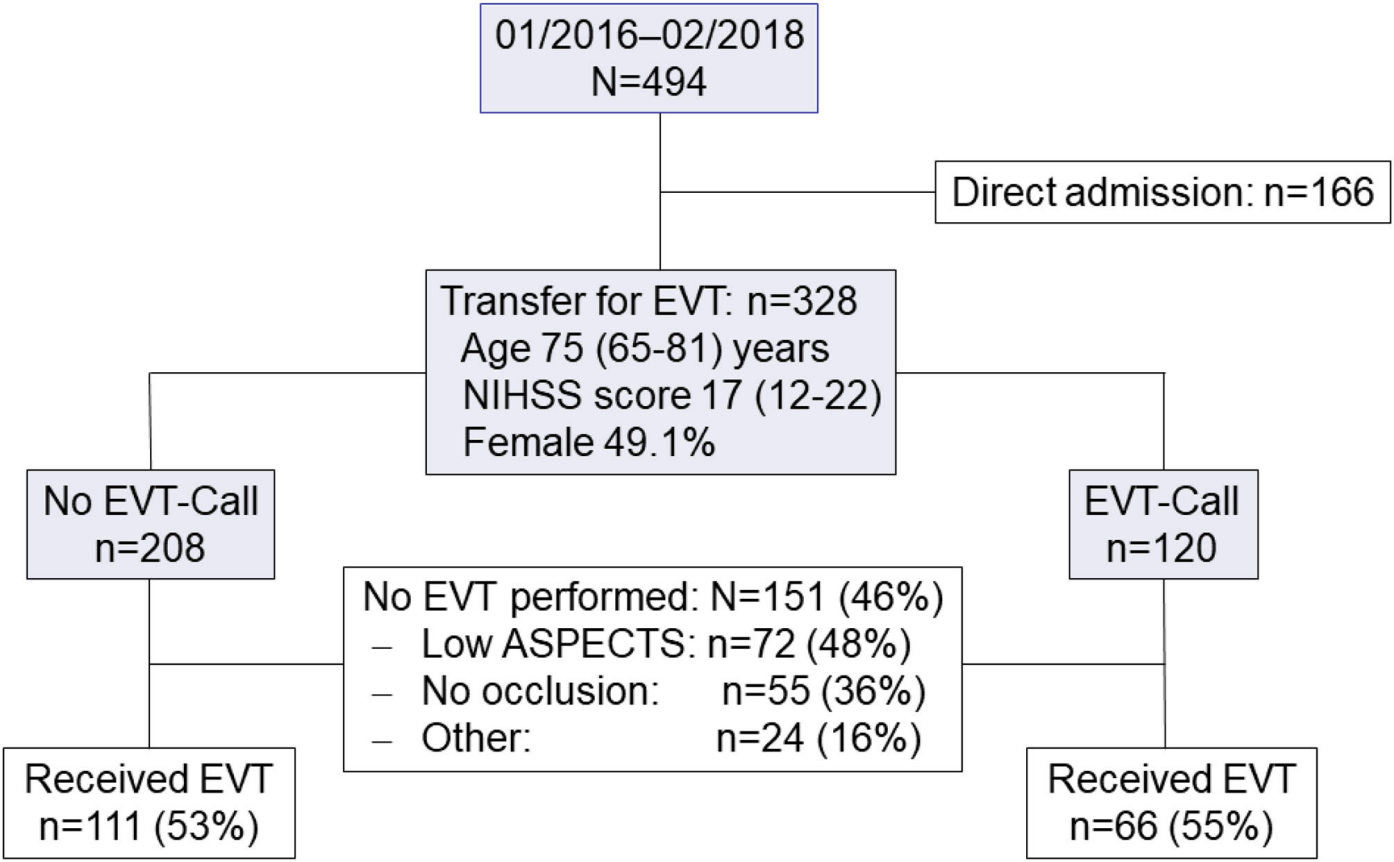
Patient flow diagram.

During the study period, we screened 494 patients for EVT at our center of whom 328 patients were transferred from remote hospitals. Of these, 120 patients presented before and 208 patients after implementation of the EVT-call (Figure 2). Clinical and imaging characteristics of patients who presented before and after implementation of the EVT-call are summarized in Table 1. The overall median (interquartile range [IQR]) age was 75 years [65–81], baseline NIHSS score 17 [12–22] and 161 patients (49.1%) were female.

**Table 1:** Clinical and imaging baseline characteristics of patients according to implementation of the EVT-call; ASPECTS indicates Alberta stroke programme early CT score; IQR, interquartile range; IVT, intravenous therapy; NIHSS, National Institute of Health Stroke Scale.

**Characteristic**	**All**	**Before EVT-call**	**After EVT-call**	***p*** **-value**
Number, *n*	328	208	120	
Age (years), median (IQR)	75.9 (65.4–81.1)	74.6 (64.0–80.1)	77.0 (68.3–82.3)	0.058
Gender female, *n* (%)	161 (49.1)	95 (45.7)	66 (55.0)	0.11
Baseline NIHSS score, median (IQR)	17 (12–22)	17 (12–23)	16.5 (12.3–19)	0.235
IVT prior to transfer, *n* (%)	221 (67.4)	146 (70.2)	75 (62.5)	0.271
Intubation prior to transfer, *n* (%)	28 (8.5)	23 (11.1)	5 (4.2)	0.026
Arterial hypertension	255 (77.7)	169 (81.3)	86 (71.7)	0.418
Diabetes	107 (30.8)	67 (32.2)	34 (28.3)	0.897
Atrial fibrillation	177 (50.3)	111 (53.4)	54 (45.0)	1
Prior stroke	43 (13.1)	25 (12.0)	18 (15.0)	0.219
Hypercholesterolemia	83 (25.3)	61 (29.3)	22 (18.3)	0.33
Current smoker	18 (5.5)	13 (6.3)	5 (4.2)	0.793
ASPECTS score, median (IQR)	6.0 (5.0–8.0)	6.0 (5.0–7.0)	7.0 (5.0–8.0)	0.171

After repeated imaging at our center, 111 patients (53%) who presented before and 66 patients (55%) who presented after implementation of the EVT-call received EVT (Figure 2). Major reasons to withhold EVT in further patients were low ASPECTS score and absence of intracranial large vessel occlusion. After implementation of the EVT-call, fewer patients were intubated prior to transfer (*p* = 0.026) (Table 1). Among the 177 patients who received EVT, 50 patients (28.2%) had a favorable outcome, 75 patients (42.4%) had an unfavorable outcome and 52 patients (29.4%) were deceased at 3 months (Table 2).

**Table 2:** Time metrics and outcome parameters according to implementation of the EVT-call; EVT indicates endovascular therapy; IQR, interquartile range; mRS, modified Rankin Scale, mTICI, modified Treatment in Cerebral Infarction.

**Characteristic**	**All**	**Before EVT-call**	**After EVT-call**	***p*** **-value**
**Number,** ***n***	328	208	120	
Door-to-image (min), median (IQR)	15 (10–20)	18 (14–22)	9.5 (7–13)	<0.001
EVT performed, *n* (%)	177 (54.0)	111 (53.4)	66 (55.0)	0.818
Image-to-groin (min), median (IQR)	52 (40–64.5)	54 (43.5–69.25)	47 (38.3–58.75)	0.042
Door-to-groin (min), median (IQR)	67 (55–82.8)	74 (58–86.5)	60 (49.3–71)	<0.001
Periinterventional intubation, *n* (%)	88 (49.7)	47 (42.3)	41 (62.1)	0.021
Groin-to-reperfusion (min), median (IQR)	63.5 (46–93.8)	66.5 (45–98.5)	61 (47–87)	0.221
Door-to-reperfusion (min), median (IQR)	133 (108.3–172)	142.5 (113.8–177.5)	122.5 (104–151)	0.018
Onset-to-groin (min), median (IQR)	298.5 (255–355)	303 (265.3–360)	275 (247.3–335)	0.023
mTICI 2b/3, *n* (%)	121 (68.4)	72 (64.9)	49 (74.2)	0.243
mRS scores 0–2, *n* (%)	50 (28.2)	34 (30.6)	16 (24.2)	0.248
Deceased, *n* (%)	52 (29.4)	32 (28.8)	20 (30.3)	0.613

### Treatment Times According to Implementation of the EVT*-*Call

**Figure 3:**
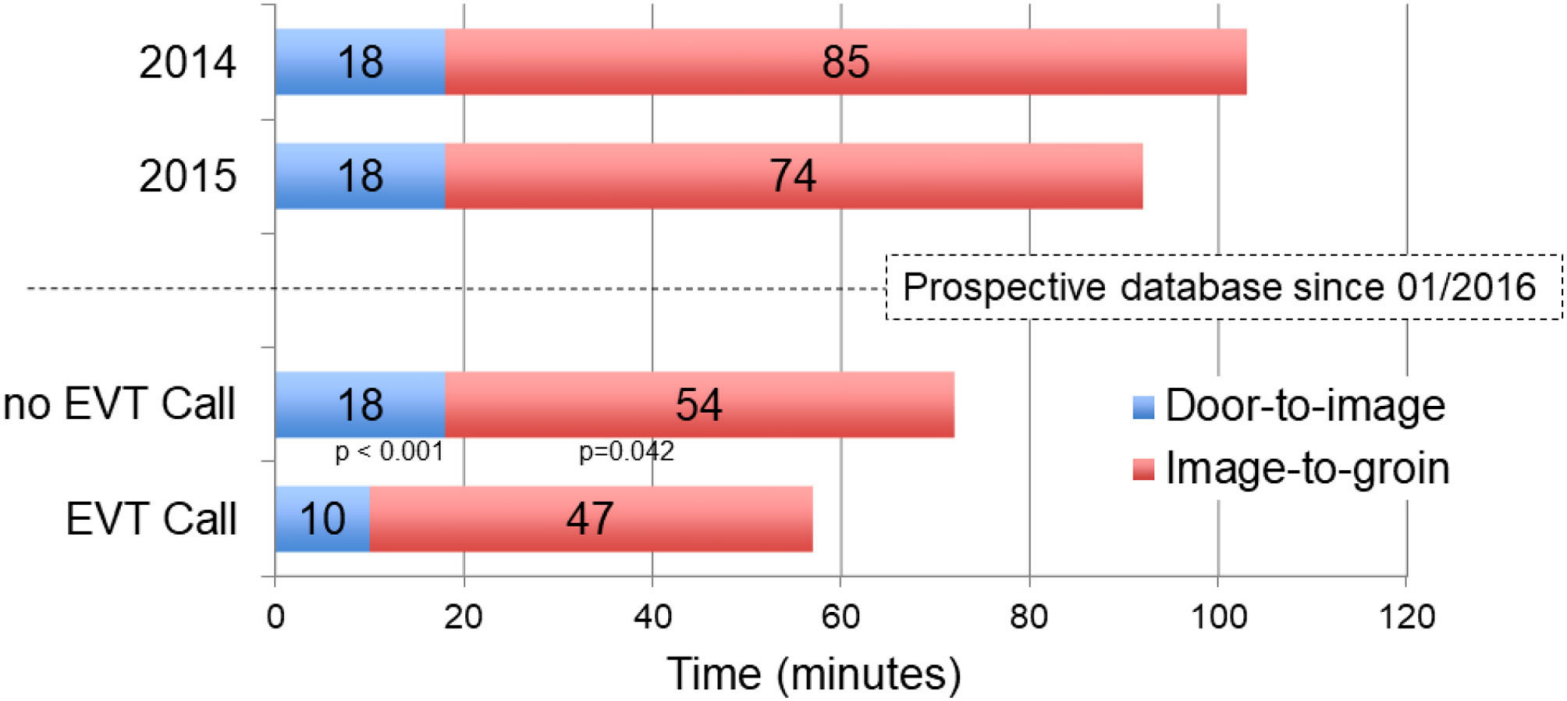
Comparison of median Door-to-image and Image-to-groin times in 2014 and 2015 and before and after implementation of EVT-call.

At the beginning of the study period in 2016, door-to-image time and image-to-groin time were already decreased before implementation of the EVT call compared to years 2014 and 2015 when we had not yet established the prospective EVT registry (Figure 3). After implementation of the EVT-call, median door-to-image time (*p* < 0.001) and image-to-groin time (*p* = 0.042) were further reduced with a resulting decrease of the door-to-groin time from 74 min (58–86) to 60 min (49–71; *p* < 0.001). Reduced treatment times had no effect on favorable functional outcome and mortality (Table 2, Supplementary Figure 1). Compared to patients who received EVT before implementation of the EVT-call, more patients received EVT with periinterventional intubation after implementation of the EVT-call (*p* = 0.026; Table 2).

The effect of the EVT-call on reduced treatment times was robust after its transfer into clinical routine with a median door-to-groin time of 63 min (52–74) in 2019 and 60 min (49–74) in 2020 despite the COVID-19 pandemia.

### Effect of EVT-Call Depending on On-Call Periods

**Figure 4:**
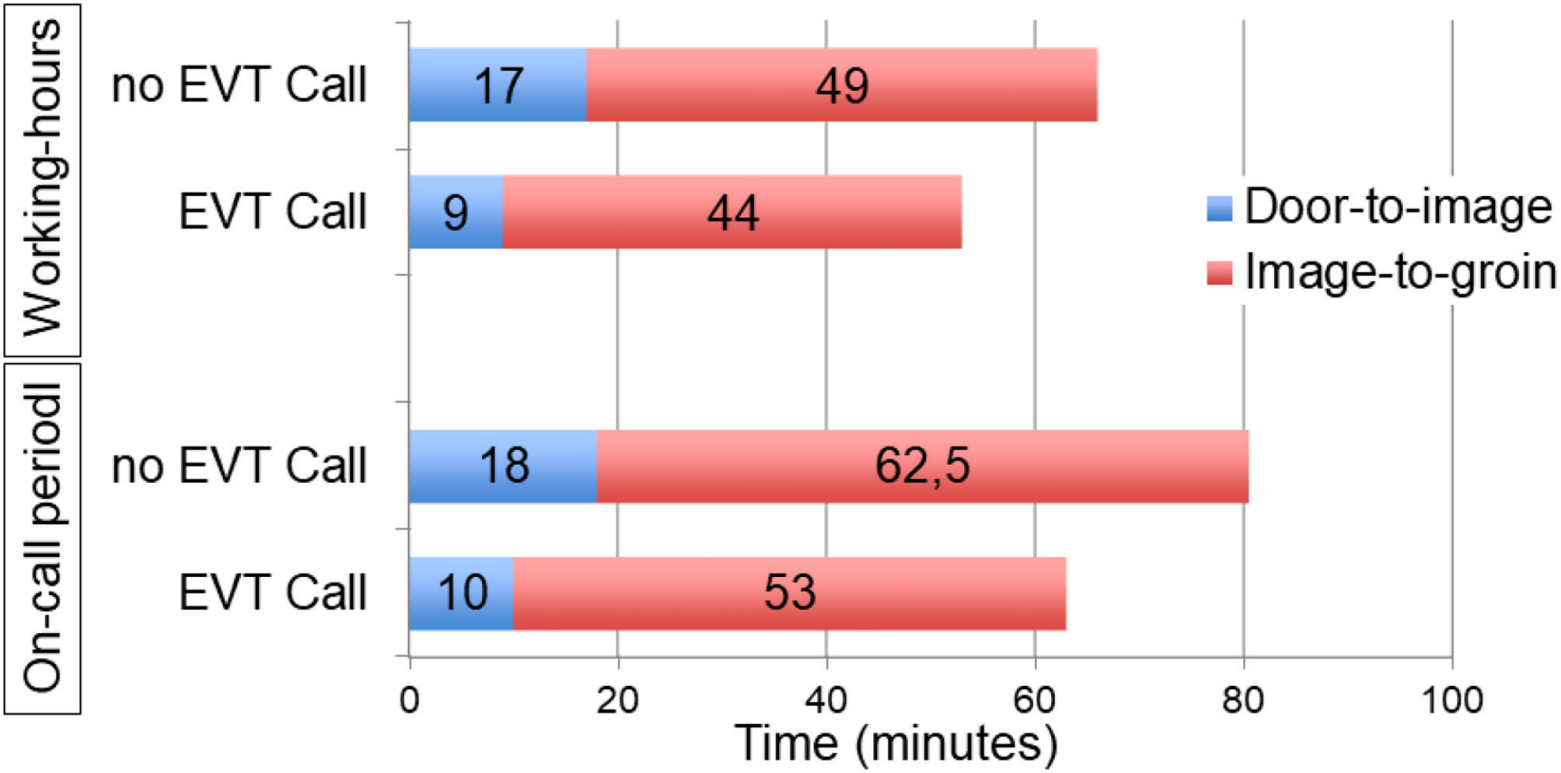
Comparison of median Door-to-image and Image-to-groin times before and after implementation of EVT-call during working-hours and on-call periods.

The effect of the EVT-call on treatment times during working hours (workdays, 7:00 a.m. to 5:00 p.m.; *n* = 122 patients) compared to on-call periods (*n* = 204 patients) is summarized in Figure 4 and Supplementary Figure 2. Median door-to-groin times were reduced from 65 (53–79) min to 54 (46–61) min during working hours compared with 83 (69–96) min to 64 (53–79) min during on-call periods without a statistically significant interaction (interaction *p*-value, *p* = 0.98).

## Discussion

Our study demonstrates that standardized team prenotification of all team members who are involved into acute treatment decision making and treatment of patients with acute ischemic stroke considered for EVT results in faster treatment times. Particularly, door-to-imaging times and image-to-groin were decreased with a resulting reduction of door-to-groin time by nearly 15 min. Time-reduction was particularly observed during on-call periods, though this interaction was not statistically significant. As a side effect, overall awareness for acute ischemic stroke as a medical emergency increased at our hospital.

Treatment times were in line with proposed time metrics from guidelines and with treatment time targets of trials with focus on rapid treatment work-flow (7, 8, 17). However, despite implementation of the EVT-call, the door-to-groin puncture time in our cohort was still longer compared to the average door-to-groin puncture time of patients transferred via a primary stroke center in the German stroke registry (median door-to-groin time: 44 min [IQR 30–67]) (18). Potential reasons for delay in our hospital are the relatively high rate of periinterventional intubation during the study period (62.1%) and repeated imaging after arrival at our center as a standardized procedure. In patients from the German stroke registry who received repeated imaging, door-to-groin times were comparable to our data (door-to-image time: 15 min [10–23]; image-to-angio time: 22 min [15–36]; angio-to-groin time: 20 min [15–31]) (18). Moreover, door-to-groin puncture times were significantly delayed in patients who received repeated imaging in a recent report from the Amsterdam stroke registry (19).

It is debatable whether repeated imaging is required for stroke patients transferred from primary stroke centers for EVT. However, in our cohort, nearly 40% of transferred patients did not receive EVT due to extensive early ischemic changes or vessel recanalization, probably secondary to long distances and transfer times in our network (11, 20, 21). In the Amsterdam stroke registry, EVT was not performed in 55% of patients who received repeated imaging—mostly due to recanalization in patients who had improved clinically (19). Novel one-stop management strategies with flat panel detector CT and CT perfusion strategies in the angiography suite may enable significant decrease of door-to-groin puncture times in patients who receive repeated imaging, but need to be tested in larger patient cohorts treated at different EVT centers (22).

Rates of periinterventional intubation may have prolonged treatment times in our center. Treatment times until groin puncture tended to be delayed in patients who received EVT under general anesthesia in the randomized GOLIATH and AnSTROKE trials (23). Whereas, randomized trial data do not point to a detrimental effect of periinterventional general anesthesia, analyses of large study cohorts suggest a negative impact on favorable outcome (23). Regardless of the direct effects of general anesthesia on functional outcome, avoidance of periinterventional intubation has potential to further reduce door-to-groin times. Furthermore, our own data has shown that prolonged general anesthesia after EVT is associated with worse functional outcome, therefore we have adapted our standard operational procedure targeting timely extubation after EVT (24).

Our faster treatment times did not translate into better outcomes measured by mRS at 3 months. It is possible that our patient population is too small to show this effect. Despite this, there is no doubt that faster treatment times generally translate into improved clinical outcomes in stroke treatment, it can be challenging to show this association outside of randomized controlled trials (25). Furthermore, only 28.2% of our patients reached a mRS of 0–2 at 3 months and 29.4% were deceased. A generally poorer outcome of stroke patients who are transferred for EVT has been described previously and may be linked to an overall lower rate of actually performed EVTs, mostly due to already progressed cerebral ischemia (11, 12, 26, 27).

Though a numerically greater time benefit of the EVT-call was observed during on-call periods (Figure 4), this interaction was not statistically significant. This may be due to overall low numbers but was unexpected. Admission on weekends was independently associated with longer door-to-groin puncture times in comprehensive stroke center patients in the German stroke registry (odds ratio 1.61; 95% CI 1.37–1.97) (18). However, despite delay of door-to-groin puncture times during on call periods, groin-to-reperfusion times and functional outcomes were unchanged in a recent study (28).

Treatment times had already decreased in 2016 compared to 2014 and 2015, potentially as a result of positive trial results with, thereafter, clear criteria for EVT (1). Though the focus of the current study was on EVT patients transferred from primary stroke centers or telestroke centers, we have recently established an intravenous thrombolysis call (IVT-call) in 2019 to decrease door-to-needle times, which also resulted in decreased median door-to-groin puncture times of directly admitted patients. As presented, the effect of the EVT-call on reduced treatment times was robust after its transfer into clinical routine even during the COVID-19 pandemic. Door-to-image times in our center remained unchanged during the first COVID-19 wave in Germany (29).

The EVT-call at our hospital is semi-automated and phone-based and delivered by a central dispatcher. To avoid an intermediary person and possible delay of information, other forms of delivery like a paging system or a secured messaging system could enhance our pre-notification procedure.

Our study is limited by its monocentric design. However, as the EVT-call itself is simple if expected transfer times are known or can be calculated, widespread external validity is likely. Centers with different patient workflow and in-hospital logistics may need to find individual solutions, e.g., presence of all team members on patient arrival may be difficult to accomplish in smaller teams. Technical solutions like electronic image analysis and smartphone image transfer may help to fasten treatment times in such scenarios. Apart from in-hospital workflow after patient arrival at the EVT center, patient selection in remote hospitals or preclinical patient selection of EVT patients and methods to speed-up transport to the intervention center are of critical importance and not addressed in this study (30, 31). The rate of intubation of patients before transfer was significantly higher before implementation of the EVT call. However, since the decision to intubate lies in the discretion of the treating physicians at the transferring hospital and is therefore not effected by our in-hospital procedures, we do not think that one can assume a causal relationship. Finally, it is difficult to determine to what extent further improvement of treatment times after implementation of the EVT-call simply resulted from increased routine. However, since most other parameters were similar before and after implementation of the EVT-call, we think that the significant faster treatment times can be mainly derived from our procedure.

## Conclusions

We have demonstrated that team prenotification with an EVT-call, which informs all team members about impending arrival and expected arrival time of patients who are potentially eligible for EVT, can decrease in-hospital treatment times and—potentially—improve functional outcomes of acute stroke patients. Door-to-groin puncture time reduction of 15 min translates into nearly 3 months healthy life-time gain, on average, emphasizing its clinical relevance (4). Several aspects that could further decrease treatment times (e.g., necessity for repeated imaging, periinterventional general anesthesia) and methods to effectively select patients in the preclinical or early clinical setting need to be analyzed in future studies.

## Data Availability Statement

The datasets generated during and/or analyzed during the current study are available from the corresponding author on reasonable request.

## Ethics Statement

The studies involving human participants were reviewed and approved by Ethikkommission an der Technischen Universität Dresden. Written informed consent for participation was not required for this study in accordance with the national legislation and the institutional requirements.

## Author Contributions

L-PP and VP contributed to the study design. MK, SW, VP, and L-PP were involved in statistical analyses. L-PP, SW, KH, JG, CH, VP, and JL were involved in data collection. L-PP, KB, JB, SW, TS, MK, HT, JR, and VP were involved in drafting the manuscript and revising it critically for important intellectual content. Each author has made substantial contributions to conception and design, or acquisition of data, or analysis and interpretation of data. All authors have given final approval of the version to be published.

## Conflict of Interest

The authors declare that the research was conducted in the absence of any commercial or financial relationships that could be construed as a potential conflict of interest.

## Publisher's Note

All claims expressed in this article are solely those of the authors and do not necessarily represent those of their affiliated organizations, or those of the publisher, the editors and the reviewers. Any product that may be evaluated in this article, or claim that may be made by its manufacturer, is not guaranteed or endorsed by the publisher.
