# Supplementary figures and images for: Team Prenotification Reduces Procedure Times for Patients With Acute Ischemic Stroke Due to Large Vessel Occlusion Who Are Transferred for Endovascular Therapy
Source: Front Neurol. 2022 Jan 3;12:787161. doi: 10.3389/fneur.2021.787161 (PMC8761669; doi:10.3389/fneur.2021.787161)

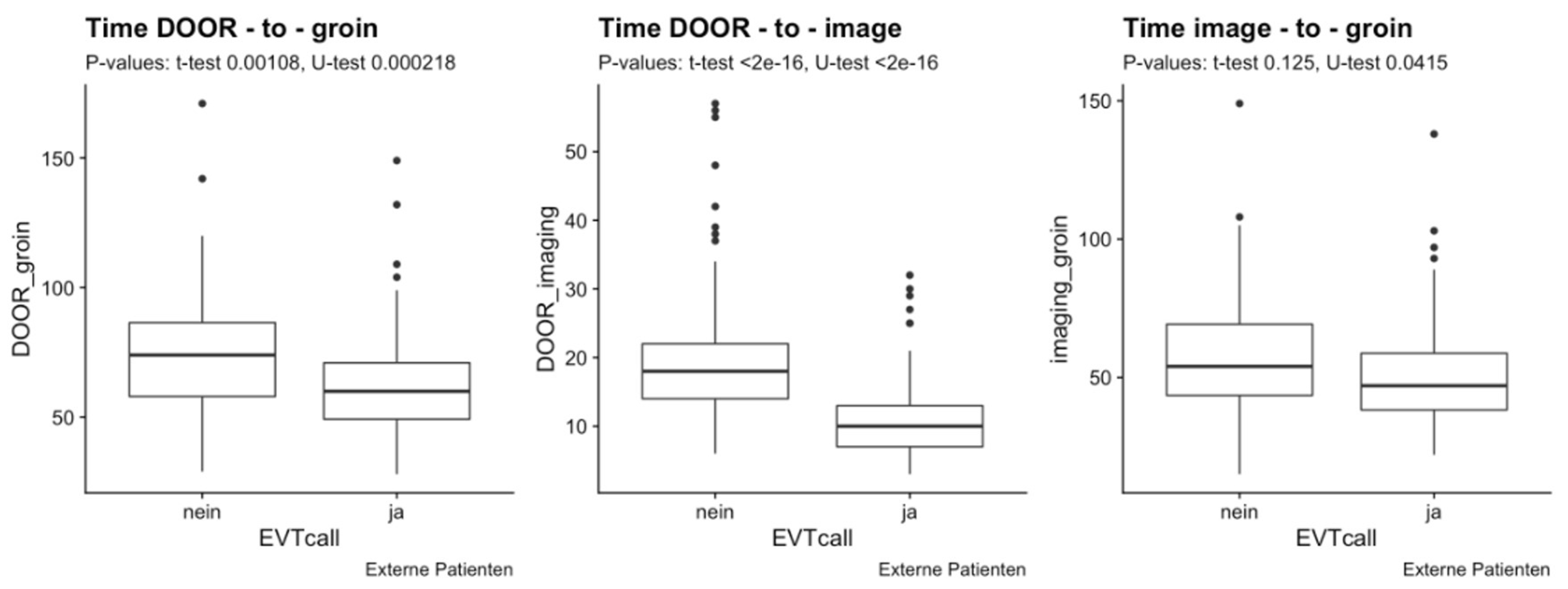

Supplement: Supplementary file 1 [file Image_1.JPEG]

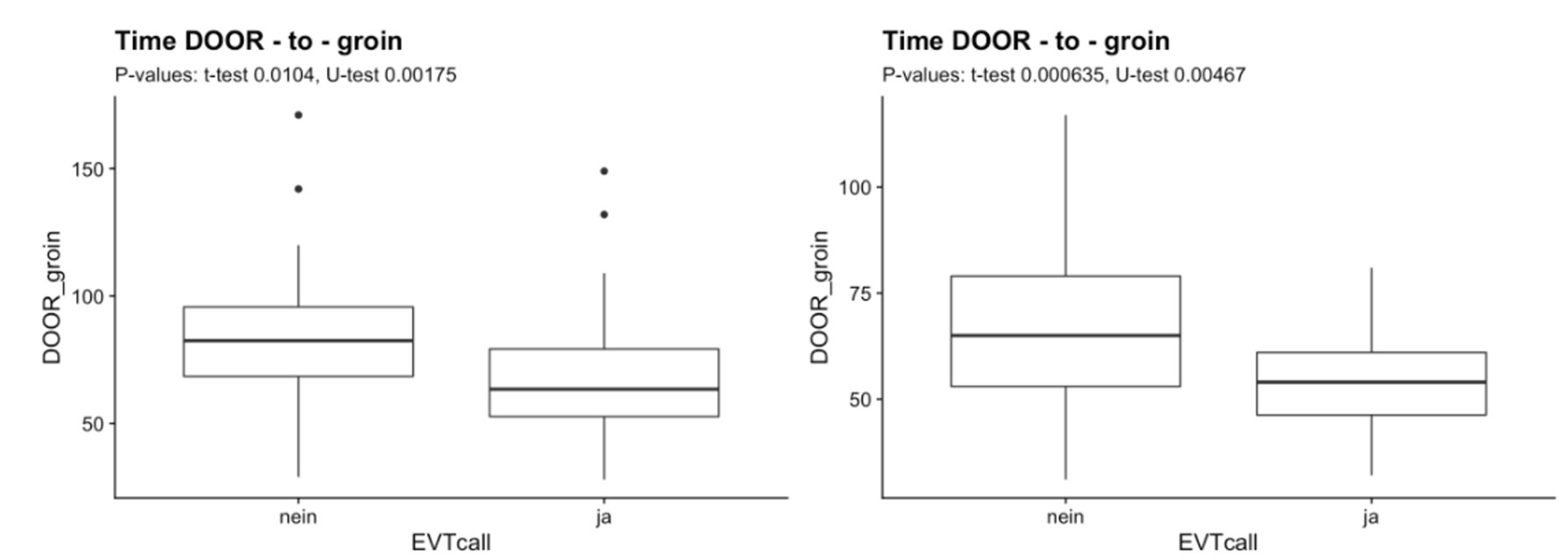

Supplement: Supplementary file 2 [file Image_2.JPEG]
